# Genome of the destructive oomycete *Phytophthora cinnamomi* provides insights into its pathogenicity and adaptive potential

**DOI:** 10.1186/s12864-021-07552-y

**Published:** 2021-04-26

**Authors:** Juanita Engelbrecht, Tuan A. Duong, S. Ashok Prabhu, Mohamed Seedat, Noëlani van den Berg

**Affiliations:** Department of Biochemistry, Genetics and Microbiology, Forestry and Agricultural Biotechnology Institute, University of Pretoria, Pretoria, South Africa

**Keywords:** Oomycete, *Phytophthora*, Invasive, Effectors, Two-speed genome

## Abstract

**Background:**

*Phytophthora cinnamomi* is an oomycete pathogen of global relevance. It is considered as one of the most invasive species, which has caused irreversible damage to natural ecosystems and horticultural crops. There is currently a lack of a high-quality reference genome for this species despite several attempts that have been made towards sequencing its genome. The lack of a good quality genome sequence has been a setback for various genetic and genomic research to be done on this species. As a consequence, little is known regarding its genome characteristics and how these contribute to its pathogenicity and invasiveness.

**Results:**

In this work we generated a high-quality genome sequence and annotation for *P. cinnamomi* using a combination of Oxford Nanopore and Illumina sequencing technologies. The annotation was done using RNA-Seq data as supporting gene evidence. The final assembly consisted of 133 scaffolds, with an estimated genome size of 109.7 Mb, N50 of 1.18 Mb, and BUSCO completeness score of 97.5%. Genome partitioning analysis revealed that *P. cinnamomi* has a two-speed genome characteristic, similar to that of other oomycetes and fungal plant pathogens. *In planta* gene expression analysis revealed up-regulation of pathogenicity-related genes, suggesting their important roles during infection and host degradation.

**Conclusion:**

This study has provided a high-quality reference genome and annotation for *P. cinnamomi*. This is among the best assembled genomes for any *Phytophthora* species assembled to date and thus resulted in improved identification and characterization of pathogenicity-related genes, some of which were undetected in previous versions of genome assemblies. *Phytophthora cinnamomi* harbours a large number of effector genes which are located in the gene-poor regions of the genome. This unique genomic partitioning provides *P. cinnamomi* with a high level of adaptability and could contribute to its success as a highly invasive species. Finally, the genome sequence, its annotation and the pathogenicity effectors identified in this study will serve as an important resource that will enable future studies to better understand and mitigate the impact of this important pathogen.

**Supplementary Information:**

The online version contains supplementary material available at 10.1186/s12864-021-07552-y.

## Background

*Phytophthora cinnamomi* (Rands 1922) is a soil-borne oomycete plant pathogen that affects natural ecosystems, nurseries, and horticultural crops worldwide. It is considered to be one of the top 10 most destructive oomycete pathogens based on the extent of economic and ecological damage it has caused [1]. While it has been observed on forest plantation trees and in natural ecosystems, the most severe economical impact has been on the horticulture industry specifically on avocado, durian, chestnut, macadamia, peach, and pineapple [2]. *Phytophthora cinnamomi* has a wide host range and has been reported to infect more than 5000 species [2], hence, referred to as the “biological bulldozer”, threatening many native plant species especially in the temperate regions of the world [3]. The most severe impact on natural ecosystems has been observed on chestnut stands in the United States of America and Europe, native oak species in Mexico and across the Iberian Peninsula, and natural vegetation in Western Australia, where 40% of almost 6000 plant species were reported to be susceptible to *P. cinnamomi* [4].

Due to the enormous economic losses and the significant impact *Phytophthora* spp. have on the environment, there has been a growing interest in the genetics and genomics of this genus [5]. The first oomycete genomes to be sequenced were that of *Phytophthora sojae* and *Phytophthora ramorum* in 2006 [6]*.* Since then the wealth of oomycete genomic data has significantly increased due to affordable next-generation sequencing technologies. This has resulted in speeding up the process of: developing diagnostic tools, resolution of evolutionary relationships, characterization of genetic variation to name but a few applications [7–9]. In addition, genomic resources have allowed for a better understanding of the biology of several oomycete pathogens. For example, phylogenetic and SSR markers were developed for *P. ramorum* based on the genome and were employed in diagnostics and diversity studies [10]. Follow up *in planta* transcriptomics helped in the identification and characterization of effectors in *P. ramorum*. The genome sequence of *P. ramorum* has also allowed comparative genomic studies to be conducted [10].

*Phytophthora* genomes are highly heterozygous with a high repetitive content and therefore pose a challenge to assemble using second generation sequencing technologies. Reports of polyploidy in many *Phytophthora* spp. further complicate the assembly process [11–13]. Third generation sequencing technologies such as Nanopore and PacBio SMRT offer improved read lengths of hundreds of kilobases, which can bridge most repetitive regions present in the genome, have proven to be useful in assembling repetitive genomes. With the use of 3rd generation sequencing contiguous genomes can now be assembled with more ease. The high error rate associated with these technologies can be overcome by making use of a hybrid approach [14]. Malar et al (2019) used PacBio, Illumina and Sanger reads to assemble the genome of *P. ramorum* and were able to improve the genome assembly from 65 Mb (2576 scaffolds) to 70 Mb (1512 scaffolds).

Oomycete species harbor a distinct set of genes that moderate host-pathogen interactions [6]. These genes encode for small-secreted proteins, such as effectors, which interfere with host defense processes. These secreted effectors act either in the extra-haustorial matrix (termed apoplastic effectors) or within the plant cells (termed cytoplasmic effectors). The most studied oomycete cytoplasmic effector proteins are crinklers (CRNs) and RxLR class effectors [15]. Crinkler proteins are present in all plant pathogenic oomycetes, whereas the RxLRs mostly occur in *Phytophthora* spp. [15, 16]. The availability of genomics and transcriptomics data has made it possible to predict putative effector homologs in *Phytophthora* spp. RxLR effectors tend to be highly diverse between species and many of these are specific to a given species. For instance, out of a large number of available effectors, only 16 RxLR-dEER effectors have orthologs in *P. infestans, P. ramorum*, and *P. sojae* [17]. As a result of their high divergence, identifying RxLR-dEER orthologs can be difficult.

Little genomic research has been done on *P. cinnamomi,* which is surprising considering the economic and ecological relevance of this species. Some notable examples include Meyer et al (2016) which performed dual RNA-Seq of susceptible *Eucalyptus nitens* plants inoculated with *P. cinnamomi* and found that the highest expressed pathogen gene *in planta* was a member of the CRN family protein (putative crinkler effector (CRN1)) [18]; Reitmann et al (2017) which identified genes expressed in vitro during the pre-infection stages and investigated the expression patterns of putative pathogenicity genes using RNA-Seq of cysts and germinating cysts [19]; and McGowin & Fitzpatrick (2017) which conducted an in silico identification of the effector arsenal and investigated their expansion and evolution in oomycete species which also included *P. cinnamomi* [20]. Currently there are five draft genome sequences available for *P. cinnamomi* [21, 22], however all of these were sequenced and assembled with only Illumina data and as a result are highly fragmented.

In the present study, we generated a high-quality reference genome for *P. cinnamomi* using a combination of Nanopore and Illumina sequencing platforms. The available and newly generated RNA-Seq data was used to assist in the annotation of the genome. Various pathogenicity effectors were identified and their *in planta* expressions investigated.

## Results and discussion

### Nanopore sequencing yielded a highly continuous assembly for *P. cinnamomi*

Nanopore sequencing using three MinION flowcells generated a total of 14.73 Gb data, with a read N50 of 12.7 kb. Illumina HiSeq sequencing generated 101.8 million 2 × 151 bp paired-end reads, of which 68.8 million paired-end reads were retained after trimming with Trimmomatic. Genome profiling with Illumina data using GenomeScope reported the sequenced isolate to be a triploid, with an expected genome size of around 107 Mb and 1.36% genome-wide heterozygosity (Figure S1). Genome assembly with Canu [for read correction] and SmartDenovo [for assembly] generated 430 contigs, with a N50 of 542.4 Kb, and a sum contig size of 126.9 Mb. After removing redundant contigs, the assembly consisted of 248 contigs, with a N50 of 629.5 kb, and a sum contig size of 107.5 Mb. Scaffolding the curated contigs with SSPACE-Longread, followed by gap filling with PBJelly and polishing with paired-end data using Pilon and Racon resulted in a final assembly of 133 scaffolds with a sum scaffold size of 109.7 Mb (Table 1). The N50 of the final assembly was 1.18 Mb, L50 was 30, and the longest scaffold was over 4.5 Mb. BUSCO (Benchmarking Universal Single-Copy Orthologs) analysis of the final assembly using the Stramenopile dataset resulted in a BUSCO score of 97.5%. Thirteen duplicated BUSCOs were identified (5.6%), while two (0.9%) were fragmented and four (1.6%) were not found. The BUSCO analysis suggests that the current assembly is highly representative of the gene space in *P. cinnamomi* and compares favorably to other available *P. cinnamomi* genome assemblies (Table S1).

**Table 1:** Characteristics of genome assembly and annotation of *Phytophthora cinnamomi*

**Assembly**	
Assembled genome size (Mb)	109.7
Gaps (Mb)	0.34
GC Content (%)	54
No. of Scaffolds	133
N50 (Mb)	1.18
L50	30
Longest scaffold (Mb)	4.55
BUSCO (%)	97.5
**Annotation**	
Number of predicted genes	19,981
Number of genes with alternative spliced variants	1188
Number of secreted proteins	1347
Mean gene length	1851
Mean exons per CDS	2.5
**Pathogenicity-related genes**	
RxLR effectors	181
Crinklers	49
NLPs	61

The main aim of this study was to generate a high-quality reference genome for *P. cinnamomi*. Currently, there are several versions of *P. cinnamomi* genome assemblies available [21, 22]. However, these assemblies are highly fragmented with the number of scaffolds ranging from 1314 to 10,084 scaffolds, N50 ranging from 10 to 264.5 Kb, and estimated genome sizes ranging from 53.69 to 77.97 Mb (Table S1). Thus, the assembly of *P. cinnamomi* presented in this study (which had 133 scaffolds, an N50 of 1.18 Mb, and an estimated genome size of 109.7 Mb) is highly continuous and by far the best reference genome available for *P. cinnamomi*. The increase in genome size observed was the result of the better-assembled repetitive regions that were probably collapsed in the other assemblies due to the use of short read sequencing technologies.

Despite the fact that nearly 100 oomycete genomes have been sequenced to date, only a handful of these have been assembled into less than 1000 scaffolds [23]. This can be attributed to the fact that oomycete genomes are highly heterozygous and contain a high amount of repetitive sequences. The best current publicly available assembly for any *Phytophthora* spp. is that of *P. sojae* with 83 scaffolds [23] and this was achieved with considerable effort by sequencing and primer walking of Fosmid and BAC libraries. Recently, several attempts have been made towards using long read technologies to sequence genomes of *Phytophthora* spp. [24–26]. These studies, together with our current work, indicated that long read technologies offers the clear advantage of producing highly continuous assemblies for oomycete genomes, which proves to be challenging when using short read data alone.

### Improved genome annotation allowed better identification of important effector genes

Braker predicted 19,981 protein-coding genes from the final assembly, 15,803 of which were expressed in the conditions investigated (in vitro and *in planta*). BUSCO analysis on the predicted proteome resulted in a BUSCO score of 96.6%, which was comparable to that obtained for the genome assembly, indicating that the annotation pipeline successfully recovered most of the gene space of the organism. Of the 19,981 proteins encoded by the genome, Blast2GO assigned gene ontology (GO) terms to 16,751 proteins and Pfam domain information to 12,646 proteins. SignalP predicted 1784 proteins to contain a signal peptide with 437 of these having a transmembrane domain.

Following the method described by McGowin and Fitzpatrick (2017), we identified a total of 181 putative RxLR effectors in the current version of the *P. cinnamomi* genome (Table S2). This number is much higher than the 68 RxLRs previously identified using the same pipeline on an annotation generated from a previous assembly [20]. RxLR effectors are important virulence factors as they have been shown to manipulate the host defenses to help establish disease. The prediction pipeline was based on three different criteria, namely the Win method, the Regex method and the Hidden Markov Model (HMM) search. Of the 181 RxLR effectors predicted, 92 met all three criteria, 45 met two criteria, and 44 met only one criterion (Table S2). Of these, 176 RxLR effectors had the signature RxLR motif, which has been hypothesized to play a role in the translocation and localization of these effectors inside host cells. Additionally, by using Basic Local Alignment Search Tool (BLAST) search [E-value 1e^-^^20^] against all known RxLR effectors from *Phytophthora* spp. [20], 498 proteins from *P. cinnamomi* showed homology to these RxLR effectors (Table S2). However, it has been suggested that a homology search could lead to the misidentification of RxLR effectors, thus we followed a more conservative approach and only considered the 181 candidate RxLR effectors identified following the method of McGowin and Fitzpatrick (2017) for subsequent analysis.

A total of 49 CRNs (45 genes, of which four have alternative transcript variants) (Table S3) and 61 necrosis-inducing proteins (NLPs) (Table S4) were also identified from the current version of the *P. cinnamomi* genome using functional assignment using Blast2GO. These numbers are higher than previously predicted for *P. cinnamomi* where only seven CRNs and 30 NLPs were identified [20]. CRN effectors have been identified in all plant pathogenic oomycete species sequenced to date. Out of the oomycete class *Phytophthora* spp. showed the largest expansion of CRNs in particular [20]. Therefore, it is plausible that CRNs could play an important role in the infection and pathogenesis of *Phytophthora* spp. Only a few CRNs were predicted to be secreted using SignalP. However, it has been suggested that some CRNs could be secreted by an unconventional protein secretion system that cannot be predicted in silico [27]. The mechanism of how NLPs work is not fully understood but we know that they have been shown to induce necrosis and also increase ethylene, phytoalexin and pathogenesis-related protein production [28, 29].

The number of RxLRs, CRNs and NLPs identified in this study is much larger than that identified by McGowin and Fitzpatrick (2017). This demonstrates that *P. cinnamomi* from this study was better assembled and annotated, which has enabled the identification of previously undetected pathogenicity-related genes. The same will be true for other gene categories of this species. The current annotation, therefore, is a better representation for *P. cinnamomi* and will be valuable for future research on characterizing and understanding the biology and pathogenicity of this important species.

### The *Phytophthora cinnamomi* genome is highly repetitive with a high abundance of transposable elements

**Fig. 1:**
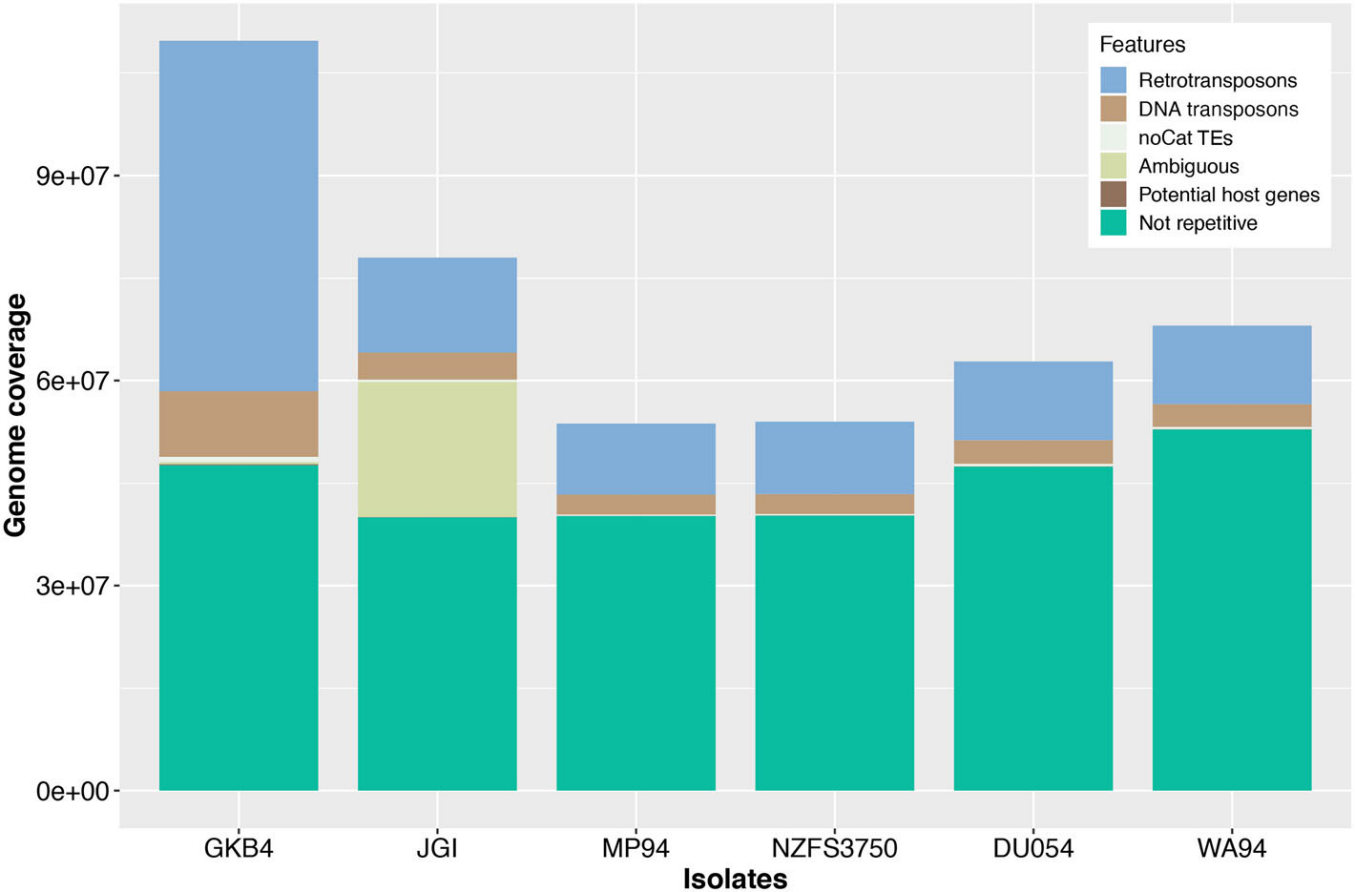
Genome size and repetitive elements from different genomes of *Phytophthora cinnamomi.* The Y-axis represents the genome size and the X-axis represents the genome assemblies of *P. cinnamomi* isolates GKB4, JGI, MP94, NZF3750, DU054 and WA94. The newly sequenced genome (isolate GKB4) had the largest genome size as a result of the expansion of transposable elements

De novo identification of transposable elements (TEs) using TEdenovo pipeline resulted in the identification of 3155 consensus TE sequences, with 2667 remaining after manual curation to remove sequences that showed homologies to known protein-coding genes. Genome annotation with this curated TE library showed that 55% of the 109.7 Mb newly assembled genome was made up of TEs. Retrotransposons were the most abundant and accounted for 35.9% of the genome space. DNA transposons accounted for 6.67% of the genome space and TEs with no classification information (noCat) accounted for 12.43% of the genome. When comparing TE coverage between different genome assemblies (Fig. 1), it was clear that the differences in assembly size observed were mainly due to the variability in TE genome coverage. The non-repetitive portion of the genome ranged from 39 to 51 Mb. The variability in non-repetitive genome sizes could be attributed to the different levels of redundancy in the assemblies as a result of different levels of heterozygosity among the isolates that were sequenced. This could also reflect the plasticity of the genomes in different isolates of *P. cinnamomi*, however, these would require high quality assembled genomes of multiple isolates for confirmation.

*Phytophthora* genomes have been shown to contain high levels of repetitive DNA sequences. The most repetitive genome characterized to date was that of *P. infestans*, in which 74% of the genome consisted of repetitive sequences [17]. *Phytophthora infestans* also has the largest *Phytophthora* genome assembled to date with an assembly size of 240 Mb. Other *Phytophthora* spp. have smaller genome sizes, and with that they also have less repetitive sequences, such as *P. capsici* (genome size of 65 Mb with 19% repetitive), *P. sojae* (genome size of 95 Mb with 39% repetitive), and *P. ramorum* (genome size of 65 Mb with 28% repetitive) [25]. With 55% of the genome made up of repetitive sequences, *P. cinnamomi* is the second most repetitive *Phytophthora* genome characterized to date. However, it is possible that many of the *Phytophthora* genomes assembled to date have incorrectly estimated genome sizes and repetitive contents as most of these genomes were sequenced using short read sequencing technologies.

### *Phytophthora cinnamomi* has a two-speed genome characteristic

**Fig. 2:**
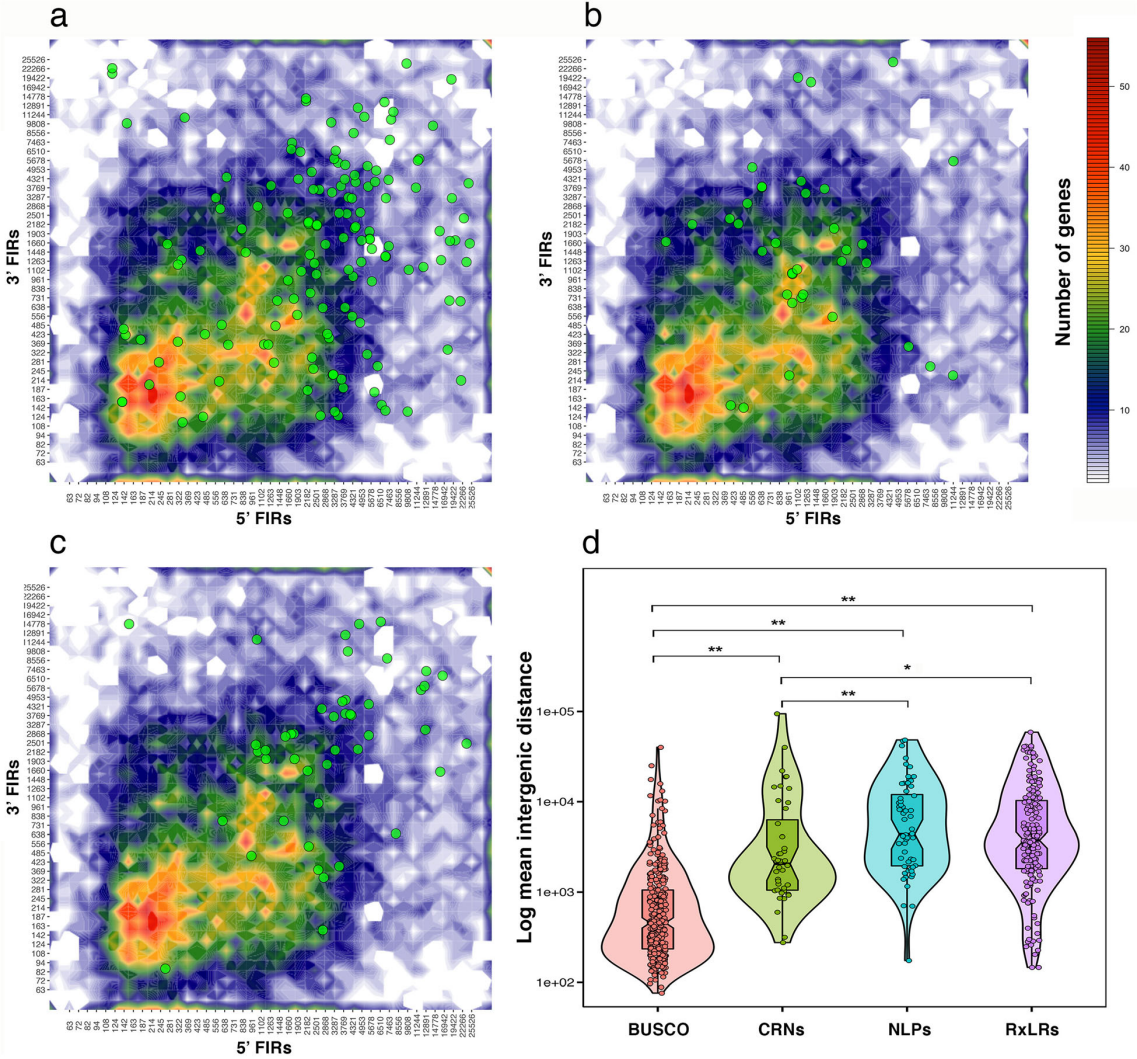
Intergenic distance analysis for three pathogenicity effector classes including (**a**) RxLRs, (**b**) CRNs, (**c**) NLPs and (**d**) comparisons between these groups of genes with the BUSCO set for Stramenopiles. Statistical significance of the difference between mean intergenic distances of different gene sets (RxLRs, CRNs and NLPs) was evaluated using the Wilcoxon rank-sum test, * denotes significance at *P* = 0.05 and ** denotes significance at *P* = 0.01

Transposable elements have been shown to play important roles in genome evolution of many plant pathogens, including oomycetes. The invasion and expansion of TEs seen in these pathogens have led to the convergent and unique patterns of genomic partitioning whereby genes important for pathogenicity and virulence tend to be found in gene-sparse and TE-rich regions, in contrast to the rest of the core genes which are found in the gene dense regions of the genome [17, 30]. This finding has led to the coin of a “two-speed genome” concept, inferring that these different genome partitions are subjected to different evolutionary rates [31]. We investigated the genomic distribution of candidate effectors genes (RxLRs, CRNs and NLPs) in *P. cinnamomi* and it was clear that these genes were also mostly found in the gene-sparse regions of the genome with increased intergenic distances (Fig. 2a, b and c). These effector genes were also found to reside in close proximity to TEs. The close distance of these genes to TEs indicate that the increased intergenic distances were due to the insertion and expansion of TEs. Statistical analysis showed that there are significant differences in the distribution of these effector genes when compared to that of the BUSCO set for Stramenopiles (Fig. 2d). This two-speed genome characteristic gives *P. cinnamomi* the potential to overcome host defense, and thus contribute to its success as a pathogen of so many plant hosts.

### *Phytophthora cinnamomi* has an overall triploid genome with varying levels of aneuploidy

**Fig. 3:**
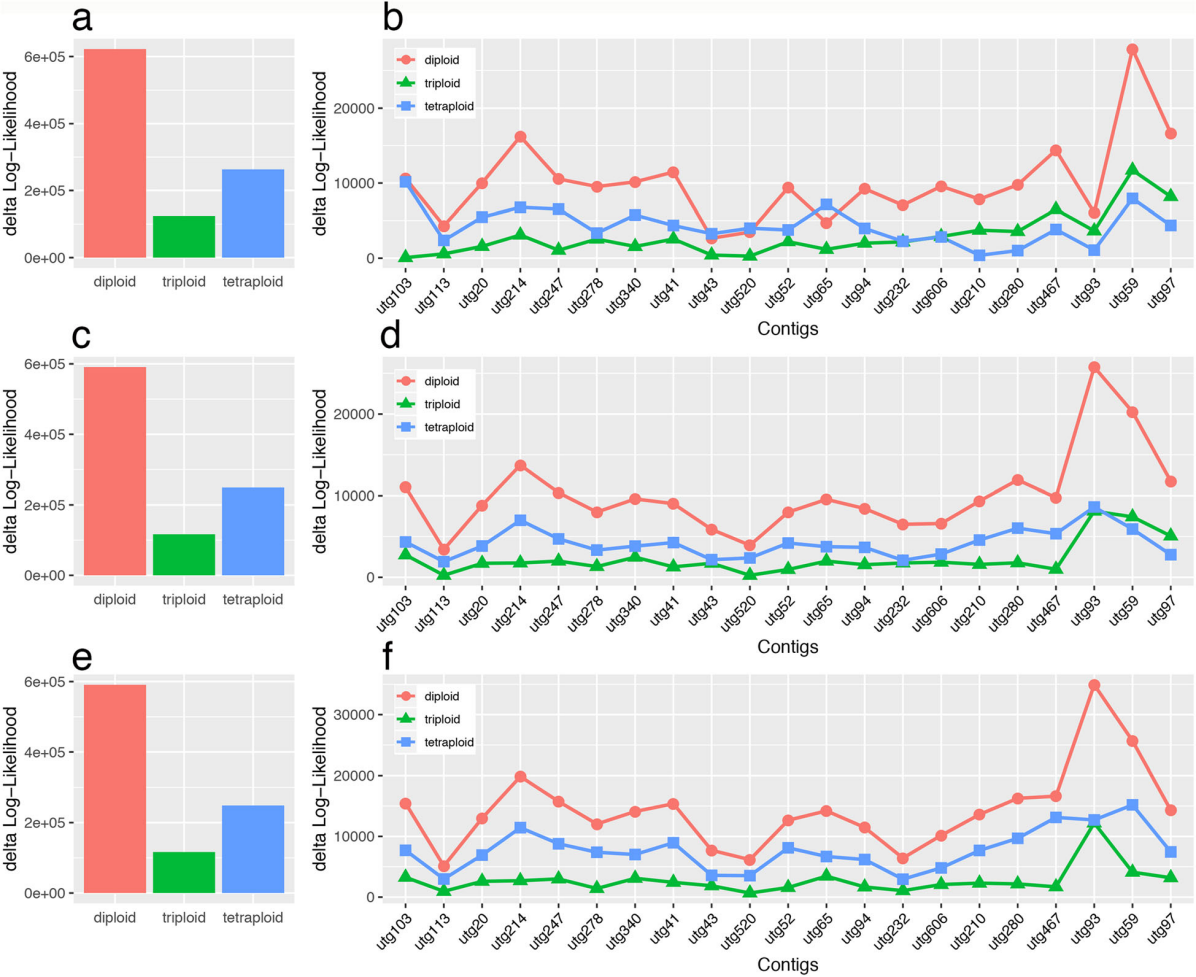
Ploidy analysis of three *Phytophthora cinnamomi* isolates (GKB4, DU054 and WA94). Ploidy analysis suggested genome-wide triploidy in GKB4 (**a**), DU054 (**c**) and WA94 (**e**). Analysis by contigs indicated tetraploidy for the last six contigs in GKB4 (**b**) and last two contigs in DU054 (**d**), whereas all the contigs investigated in WA94 indicated triploidy (**f**)

Ploidy estimation using nQuire indicated that *P. cinnamomi* sequenced in this study is a triploid at the genome level, although some scaffolds showed evidence of possible tetraploidy (Fig. 3). Similar results were also observed with two other isolates of *P. cinnamomi* (DU054 and WA94) for which Illumina data were available [22]. Ploidy analysis could not be done for the two other isolates of *P. cinnamomi* (NZFS3750 and MP94–48) due to the lack of sufficient Illumina data coverage [21]. This triploidy observed is not uncommon as this has also been reported in *P. infestans*, although *P. cinnamomi* has always been considered to be a diploid organism [32]. The presence of more than two alleles per locus has also been observed in other *Phytophthora* spp. such as *P. infestans*, *P. nicotianae* and *P. ramorum* [11, 12, 33]. The ploidy of *P. infestans* has been studied more in depth and it has been shown that this species show varied levels of ploidy including trisomy, aneuploidy and polyploidy [13, 34, 35]. Genome profiling of the sequenced isolate using GenomeScope also suggested an overall genome triploidy with a heterozygosity level of 1.36% (Figure S1). This level of heterozygosity is in the higher range compared to that from other oomycete species reported such as *P. infestans* which was 0.695% [23] and *Bremia lactucae* which ranged from 0.77 to 1.29% [36].

*Phytophthora cinnamomi* has two mating types, of which the A2 mating type is responsible for the widespread damage that has been observed. Numerous studies could not find any evidence of sexual reproduction for this species [37–40]. With the lack of sexual reproduction, it would be assumed that asexual lineages are temporary and short-lived. In the case of many *Phytophthora spp*. including *P. cinnamomi*, however, the asexual lineages have managed to become very successful and widespread. To this end, polyploidization has been suggested to help explain the success of clonal asexual lineages [41]. In *P. infestans* for example, two recent studies have shown that progenies from a sexual reproductive population were diploid whereas isolates from dominant asexual lineages that have caused the most significant damage in the past few years were found to be triploid [41, 42]. It is possible that *P. cinnamomi* also uses polyploidization as an adaptive strategy. This could also partly explain why sexual reproduction has not been observed.

### Genes involved in pathogenicity are under diversifying positive selection

The availability of draft genome sequences of *P. cinnamomi* isolates from various geographic regions and hosts offers the opportunity to identify genes under positive selection in this species. We used gKaKs pipeline [43] to calculate the dN/dS ratio for all the genes present in the current annotation by comparing sequence variation against four other published genomes of *P. cinnamomi* [21, 22] and also the *P. cinnamomi* genome available at the JGI genome portal (https://genome.jgi.doe.gov/). A total of 1184 genes (5.9% of all genes encoded by the genome) were identified to have dN ≥ 0.01 and 1 < dN/dS < 10, suggesting that these genes were under positive selection. Of these 1184 positively selected genes: 41 were RxLR effectors (22.6% of total RxLR effectors) (Table S2), 10 were CRN effectors (20.4% of total CRN effectors) and seven were NLPs (11.5% of total NLPs). The high proportion of positively selected effector genes, especially RxLRs and CRNs, compared to the 5.9% overall genome average suggests that these effector genes might be involved in the arms race between the pathogen and its hosts, which could explain the high selective pressure. These positively selected effector genes would be good candidates for functional characterization studies to understand their roles in the infection process.

### *In planta* RNA-Seq analysis reveals differential gene expression of important genes involved in pathogenesis

Differential expression analysis of RNA-Seq data obtained from *in planta* infection and in vitro mycelial growth (Table S6) identified 3328 differentially expressed genes with log fold changes ≥2 and adjusted *P*-values ≤0.01, 2141 of these were up-regulated and 1187 were down-regulated during infection (Table S7). GO analysis of up-regulated genes identified 34 molecular functions, of these hydrolase activity (GO:0004553, GO:0016798), transmembrane transporter activity (GO:0022857) and oxidoreductase activity (GO: 0016491) were significantly enriched (Figure S2). A total of 56 biological processes were identified, in which carbohydrate processes (GO:0005975, GO:0016052) and oxidation-reduction processes (GO:0055114) were among the enriched GOs (Figure S2). Many of these enriched processes are related to the infection process. For example, pectin is one of the main components of the plant cell wall and one of the enriched GO terms was pectin esterase activity (GO:0030599). We also found pathogenesis (GO:0009405), transmembrane transport (GO:0055085) and transmembrane transporter activity (GO:00022857) among the enriched GO terms.

**Fig. 4:**
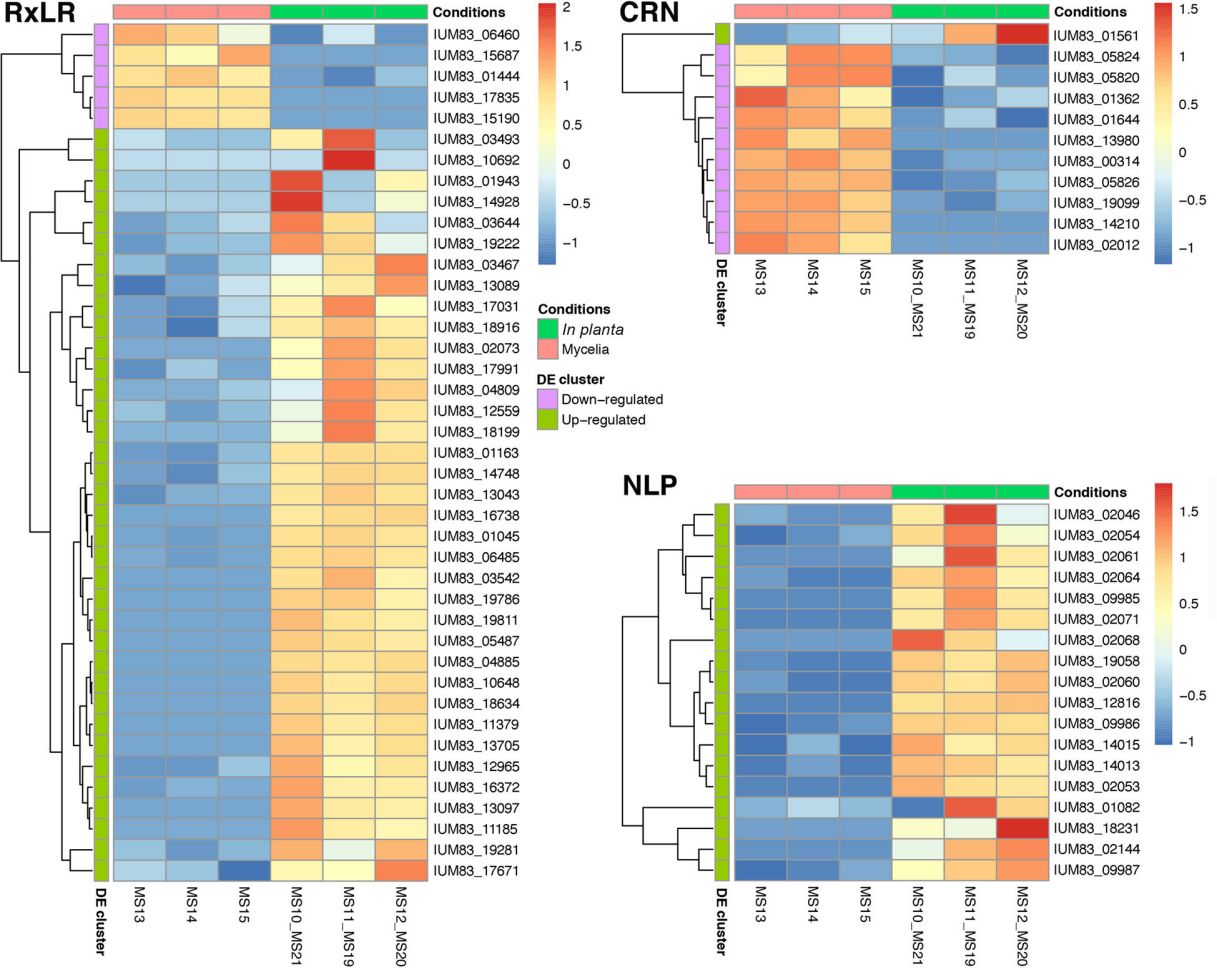
Differentially expressed RxLRs, CRNs and NLPs identified between mycelia (MS13, MS14 and MS15) and *in planta* infection (MS10_MS21, MS11_MS19 and MS12_MS20). Heatmaps were generated using the Pheatmap package. Colour gradients indicate the Z scores of the normalized counts transformed using the varianceStabilizing Transformation function in DESeq2 (Love et al., 2014)

A high proportion of effector genes (RxLRs, CRNs, and NLPs) were found to be present among the genes differentially expressed during infection. A total of 41 out of the 181 identified RxLRs (36 up-regulated and 5 down-regulated), 18 out of 61 identified NLPs (all up-regulated), and 11 out of 49 identified CRNs (1 up-regulated and 10 down-regulated) were found in the gene set (Fig. 4). Up-regulation of a high percentage of RxLRs and NLPs were expected as these genes have been shown to be important in the infection process in other *Phytophthora* spp. [44]. The up-regulation of RxLR and NLP genes during infection has also been shown in other plant pathogens [44–46]. With CRNs, however, a great proportion of the predicted CRNs showed down-regulation at 5 dpi. Only one of the predicted 11 CRNs was up-regulated. This is not surprising as it has been shown earlier that CRNs were mostly expressed in the early stages of infection, within the first 48 h [47]. It is noteworthy to mention that a high level of variability in expression data was observed among the biological replicates for some genes (Fig. 4) and this was more evident in the *in planta* samples. This variability is most probably a result of the low pathogen read count in these samples (Table S6), which is typically expected for a dual RNA-Seq experiment.

### Carbohydrate-active enzyme analysis

Carbohydrate-active enzymes (CAZymes) have been shown to play important roles in plant-pathogen interactions [48]. Analysis with dbCAN identified 468 CAZymes (including isoforms) in *P. cinnamomi* proteome (Table S5). Of these, 359 CAZymes were putative cell wall degrading enzymes (CWDEs), classified into 31 glycoside hydrolases (GHs), three carbohydrate esterases (CEs), three polysaccharide lyases (PLs), two carbohydrate-binding modules (CBMs) and one auxiliary activities (AAs) families. Of the 359 CWDEs identified, 215 had a signal peptide at the N-terminus. A total of 156 CAZyme genes were found to be up-regulated during *in planta* infection, and most of these (121) belonging to GH families. 141 of these up-regulated CAZymes were putative CWDEs. Ony 17 CAZymes were found to be down-regulated, of which 12 were putative CWDEs.

Many CWDEs are involved in the breakdown of β-1,3-glucans. The deposition of β-1,3-glucans (callose) by plant is an essential component of the primary defense response [49], and hence the pathogen’s capability to degrade callose could be an important indicator of pathogenicity. The *P. cinnamomi* genome contains 12 GH72 and 18 GH81 proteins that are known to play a role in the degradation of β-1,3-glucans. Of these four GH72 and eight GH81 were found to be significantly up-regulated during *in planta* infection. Interestingly the opposite has been found in fungal phytopathogens where low numbers (1–8 GH72 and 0–3 GH81) of these proteins have been identified [50, 51]. Unlike fungi, the cell wall of *Phytophthora* spp. is predominantly made up of β-1,3-glucans, it is therefore possible that some of these β-1,3-glucanases could also be involved in the pathogen cell wall development and modification. It has also been suggested that these larger numbers of β-1,3-glucanases give *Phytophthora* spp. an advantage in that they are better armed to degrade callose deposited by the plants as a defense response at the infection site [52].

The breakdown of pectin is one of the first events to occur in a series of CWDE activities [53]. It is thought that the breakdown of pectin increases cell wall porosity and in the process exposing other cell wall polysaccharides to other CWDEs [52]. A range of pectin-degrading enzymes are needed to degrade the wide variety of pectins present within plant cell walls [54]. Of the pectin-degrading enzymes in *P. cinnamomi*, 45 are PLs and 32 are CEs. Similar numbers of these have also been found in other *Phytophthora* spp. [52].

## Conclusion

In this study, we generated a high-quality reference genome sequence and annotation for *P. cinnamomi.* The highly continuous assembly was achieved using a hybrid approach that made use of Nanopore data for assembly and Illumina for polishing steps. Genome annotation was generated using supporting transcript evidence from RNA-Seq data. The assembly indicated that *P. cinnamomi* has a much larger genome size than what is currently estimated based on previous draft versions that were assembled only with short read data. The larger assembly size was due to a better assembly using long reads that helped resolve repetitive regions, which consisted mostly of TEs. Investigating the genome architecture indicated that *P. cinnamomi* has a typical two-speed genome signature, similar to that of *P. infestans*. The improved assembly and annotation have enabled us to better identify and characterize various pathogenicity-related genes present in the genome, which otherwise would not have been possible using previously available draft genome sequences. *In planta* RNA expression analysis identified a number of pathogenicity genes that were up-regulated during infection, which will be important for future functional characterization studies. The genome sequence of *P. cinnamomi* and its annotation generated in this study will serve as an important foundation for future genomics and genetics studies aimed at a better understanding of the biology and pathogenicity of this important species.

## Methods

### DNA extraction and genome sequencing

#### DNA extraction

*Phytophthora cinnamomi* isolate (GKB4) isolated from infected avocado roots collected from Groenkloof Block 4, Westfalia Estate, Tzaneen, Limpopo province, South Africa was selected for genome sequencing. A single hypha culture was grown in Yeast malt broth (2% yeast extract, 0.5% malt extract) for 5 days, after which mycelia was harvested, freeze-dried, and ground into a fine powder in liquid nitrogen. Around 50 mg of grounded mycelia were dissolved in 5 ml digestion buffer (10 mM Tris HCl, pH 7.9, 20 mM EDTA, 500 mM Guanidine-HCl, 200 mM NaCl, 1% Triton™ X-100) supplemented with 0.5 mg/ml cellulase and 0.5 mg/ml of lysing enzymes from *Trichoderma harzianum* (Sigma-Aldrich, St. Louis, MO, USA) and incubated at 42 °C for 2 h. After the first incubation step, RNase A (20 μg/ml) was added and the mixture was incubated for 30 min at 37 °C. Post RNase treatment, proteinase K was added (0.8 mg/ml) and the mixture was incubated for 2 h at 50 °C with gentle agitation. The mixture was centrifuged for 20 min at 7500 x g. The supernatant was collected and DNA was purified using a QIAGEN G-20 genomic-tip (Qiagen, Valencia, CA, USA) following the manufacturer’s protocol. Extracted DNA was stored at 4 °C until used for sequencing.

#### Nanopore sequencing

Nanopore sequencing was conducted using a MinION sequencing device. Three MinION flow cells (FLO-MIN106) were used for sequencing. For the first two flow cells, libraries were prepared using the SQK-LSK108 kit, and for the 3rd flow cell SQK-LSK109 kit was used. Nanopore sequencing was performed for 48 h with a MinION Mk1B sequencer. Base calling of resulting raw FAST5 files were performed using the Oxford Nanopore Albacore software (v0.8.4). Sequencing adapters were removed using Porechop (https://github.com/rrwick/Porechop).

#### Illumina sequencing

Illumina sequencing was carried out by Macrogen Inc., Korea. One paired-end library (350 median fragment size) was constructed using the TruSeq DNA PCR Free kit and sequenced on one lane of the Illumina HiSeq X Ten sequencer to obtain 151 bp paired-end reads. Read quality was assessed using FastQC tool (https://www.bioinformatics.babraham.ac.uk/projects/fastqc/), and trimmed using Trimmomatic v.0.36 [trimming parameters: ILLUMINACLIP:TruSeq-PE.fa:2:30:12 LEADING:20 TRAILING:20 SLIDINGWINDOW:4:25 MINLEN:100] to remove remaining Illumina adapters as well as bases of low quality [55].

### RNA sequencing

RNA sequencing was conducted using RNA isolated from both in vitro (mycelia) and *in planta* infection. For the in vitro experiment, fungal mycelia grown on V8 agar for 5 days were harvested and used for RNA extraction. For *in planta* infection, a zoospore suspension was used to inoculate the roots of clonal avocado plantlets provided by Westfalia Technological Services (Tzaneen, Limpopo, South Africa). Preparation of zoospore suspension was done using the method described in [56]. The roots and lower stem of two avocado rootstocks (Dusa®- partially resistant and R0.12- susceptible to *P. cinnamomi*) were submerged for 2 h in the zoospore suspension at a concentration of 1.4 × 10^5^ zoospores/ml, after which they were transplanted into 1.5 l plastic bags filled with a mixture of perlite and vermiculite (1:1). Once transplanted, the zoospore suspension that was used to infect was divided into even portions and added to the growth media of treated plantlets (50 ml/plantlet). Root material from three plantlets per rootstock was harvested 5 days post inoculation (dpi), snap-frozen in liquid nitrogen and ground to a fine powder with a homogenizer (IKA A11 basic Analytical mill; United Scientific [Pty.] Ltd.), and stored at − 80 °C until RNA extraction.

The CTAB extraction method was used for RNA extraction from both mycelia and root powders [56]. RNA concentration was determined using a Nanodrop ND-100 Spectrophotometer (Nanodrop Technologies Inc., Montchanin, DE, USA) and RNA integrity was assessed using agarose gel electrophoresis. DNase treatment of extracted RNA (1 μg) was performed by the addition of 1 U RNase-free DNase (Fermentas Life Sciences, Hanover, MD, USA), 1 μl 10x reaction buffer, and diethylpyrocarbonate-treated water to a final volume of 9 μl. The mixture was incubated at 37 °C for 30 min followed by the addition of 25 mM EDTA and incubation at 65 °C for 10 min. DNase-treated RNA was column purified using the RNeasy® MiniEluteTM Cleanup kit (Qiagen, Valencia, CA, USA) according to the manufacturer’s instructions. The concentration and integrity of total RNA were assessed with an Agilent 2100 Bioanalyzer (Agilent Technologies, California, USA) (RIN values ≥ 6.5). Purified RNA was sent to Novogene (Novogene Corporation Inc) for sequencing on the Illumina HiSeq with PE150 mode.

### Genome assembly

To estimate genome size, ploidy and heterozygosity, genome profiling using Illumina short read data was performed using GenomeScope v2.0 with a k-mer size of 21 [57]. Nanopore reads that were longer than 5 kb were selected for error correction using Canu v1.7.1 [58, 59] using an expected genome size of 125 Mb and output read coverage set at 200. Corrected reads obtained from Canu were assembled using SMARTdenovo (https://github.com/ruanjue/smartdenovo) with default settings. Contigs obtained from SMARTdenovo were polished with NanoPolish (https://github.com/jts/nanopolish) using the raw Nanopore fast5 data. After polishing, redundant contigs were removed using Purge Haplotigs [60]. The curated contigs were further assembled into scaffolds using SSPACE-Longread version 1.1 [61] with Canu corrected reads as input reads. Gaps in scaffolds were filled or extended using PBJelly version 15.8 [62] using Canu corrected reads. For further improvement of the long read assembly, trimmed Illumina data were mapped to the assembled scaffolds to generate a bam file, and this bam file was used as input to polish the assembly using Pilon [63]. Three consecutive iterations of Pilon were conducted. Finally, the pilon polished assembly was subjected to a final round of polishing with Illumina data using Racon [64]. The BUSCO tool v3.0.2 was used to assess completeness of the final assembly as well as outputs of intermediate stages of the assembly process using the Alveolata_Stramenopiles_ensembl dataset [65].

### Transposable element identification and characterization

Transposable elements were identified using TEdenovo pipeline (http://urgi.versailles.inra.fr/index.php/urgi/Tools/REPET), which uses a combination of tools (BLASTER, RECON, GROUPER, and PILER) to identify and classify TEs present in the genome [66–69]. The TE families classified as Unknown (noCat) or Potential Host Genes by REPET were subjected to manual curation by subjecting the data to a BLAST analysis against the SWISS-PROT and non-redundant protein databases. Any TE family that resulted in positive hits to non-TE-associated proteins was not considered to be TEs and hence removed from the final curated TE library. TEannot pipeline, which employs REPEATMASKER (http://repeatmasker.org), BLASTER and MATCHER were used to annotate and calculate TE coverage in the newly assembled genome of *P. cinnamomi* using the curated TE library [66]. The same TEannot pipeline and curated TE library were also used to calculate TE coverage in the other assemblies currently available for *P. cinnamomi*.

### Genome annotation

Structural annotation was conducted using Braker2 [70–72], with GeneMark and Augustus as gene predictors. The assembly was soft-masked against the curated TE library before it was used for annotation. Available RNA-Seq data from *P. cinnamomi* germinating cysts [19], as well as RNA-Seq data generated in this study from mycelia, and *in planta* infection were used as supporting gene evidence during the annotation process. First, HISAT2 [73–75] was used to align the RNA-Seq data to the masked genome. The alignment was filtered to keep only concordant paired-end reads and the filtered alignment was used for GeneMark training as part of the Braker2 pipeline. Additionally, available proteomes from four closely related *Phytophthora* spp. to *P. cinnamomi*, namely *P. sojae* [6]*, P. rubi - PRJNA244739, P. pisi - PRJEB6298,* and *P. fragariae* [76], were aligned to the genome using GenomeThreader [77] and used as additional evidence for Augustus training and prediction steps as part of the Braker2 pipeline. Functional annotation was carried out using Blast2GO plug-in in CLC Genomics Workbench [78] with a homology search against the nr database using BLASTp [E-value of 1e^− 05^] [79] and domain search using Interproscan [80].

### Effector identification

Three different classes of effectors including RxLR, CRN, and NLP were identified from the predicted proteome. These effectors have been shown to play important roles in infection and pathogenesis of *Phytophthora* spp. RxLR effectors were identified according to the methods described by McGowin and Fitzpatrick [20]. Briefly candidate effectors had to meet at least one of the three predefined methods namely: (I) Win method where the proteome was examined for the presence of a signal peptide within the first 30 amino acid residues followed by an RxLR motif between residues 30 and 60 [81]; (II) Regex method where the secretome was searched for the presence of an RxLR motif within 100 residues after the signal peptide cleavage site and the presence of an EER motif within 40 residues downstream of the RxLR motif (allowing replacements of E to D and R to K) [82]; (III) Hidden Markov Model (HMM) built from previously identified RxLR effectors from *P. ramorum, P. sojae* and *P. infestans* [17, 82, 83]. CRN and NLP genes were identified based on functional assignment by Blast2GO, which is based on homology evidence to known CRNs and NLPs as well as on the presence of Pfam domains associated with these classes of effectors.

### Carbohydrate-active enzymes and cell wall degrading enzymes

CAZymes were identified using dbCAN2 [84] using the CAZyme database classification [85]. Three different approaches were used namely DIAMOND, HMMER and Hotpep. A protein was classified as a CAZyme if it met at least two of the three criteria. Additionally, putative CWDEs were also identified as those containing glycoside hydrolase (GH), polysaccharide lyase (PL), carbohydrate esterase (CE), carbohydrate-binding module (CBM) and auxiliary activity (AA) modules that are known to be associated with the degradation of plant cell wall carbohydrates [52].

### Genomic architecture analysis

Analysis of intergenic distances was carried out as described in Saunders et al. [86]. Briefly, the 5′ and 3′ intergenic distances for all genes as well as the identified RxLRs, CRNs and NLPs were 2-dimensionally binned and plotted using GenomicRanges, rtracklayer, Rsamtools, and ggplot2 R packages. The Wilcoxon rank-sum test was used to test the statistical significance of the difference between mean intergenic distances of effector gene sets (RxLR, CRN and NLP) and the BUSCO gene set for Stramenopiles (*n* = 234).

### Identifying genes under selection

Genes under diversifying positive selection were investigated using gKaKs pipeline [43]. gKaKs computes the substitution rates (Ka [dN], Ks [or dS] and Ka/Ks [or dN/dS]) between a well-annotated genome and a non-annotated genome while taking into account frame shift mutations and premature stop codons. The genome assembly generated in this study was used as the reference genome and all five available assemblies for *P. cinnamomi* from public databases were used as target genomes to identify genes under positive selection. To avoid overestimation of dN/dS value due to a small dS value, we removed genes with dS < 0.01 from the calculation. We also removed genes with abnormally high dN/dS value (> = 10) as these could be due to alignment errors. Genes were identified to be under positive selection if it had a 1 < dN/dS < 10.

### Ploidy analysis

To estimate the ploidy level based on short read data, we used nQuire which is a statistical approach for ploidy estimation based on the distribution of base frequencies at variable sites [87]. Available short read data for two additional isolates namely DU054 and WA94 [22] were also included in the analysis. Briefly, nQuire uses the Gaussian Mixture Model (GMM)-based method to estimate ploidy. Sequenced reads are mapped to a reference genome and then base frequencies are calculated at variable sites. The likelihood is maximized using an Expectation-Maximization (EM) algorithm for both the free and the three fixed models (diploid, triploid and tetraploid). The results show a possible final state of the GMM under the assumptions of each of the four models. The *Δ*log*L* is calculated between the free model and each of the three fixed models. The fixed model with the smallest *Δ*log*L* is most likely the ploidy of the genome investigated. The ploidy was estimated for the overall genome as well as for the 21 largest contigs that were larger than 1 Mb.

### Differential gene expression analysis

In order to gain insight into genes involved in infection and host penetration, differential gene expression analysis was conducted between RNA-Seq data obtained from in vitro mycelial growth (MS13, MS14 and MS15; Table S6) and *in planta* infection (MS10, MS11, MS12, MS19, MS20, MS21; Table S6). Due to the low amount of *P. cinnamomi* reads obtained for *in planta* infection (Table S6), RNA-Seq data obtained for two different avocado rootstocks (Dusa® and R0.12) were combined and treated as a single experiment, however, keeping three replicates for statistical analysis and these were referred to as MS10_MS21, MS11_MS19 and MS12_MS20. RNA-Seq reads were first mapped to the assembly using HISAT2 [88]. In case of *in planta* RNA-Seq data, reads were mapped simultaneously to both the *P. cinnamomi* and avocado genomes [89]. Reads mapped to gene features (exons) were counted for each gene using featureCounts program [90]. Count results were imported into R and gene-level differential gene expression analysis was conducted using DESeq2 [91]. Genes with total read counts of less than 10 across all conditions were discarded. Differential expression analysis based on the negative binomial distribution was conducted using the DESeq function as part of the DESeq2 package. Genes were identified as being differentially expressed if false discovery rate (FDR) adjusted *p* values were equal to or below 0.01 and also had log fold-changes of at least 2 or higher. To generate the heatmaps of differentially expressed genes for RxLRs, CRNs and NLPs, the variance stabilizing transformation (VST function in DESeq2) was applied to the normalized count data, the Z-score values were calculated from the transformed data and used for drawing of the heatmaps using the Pheatmap package [92]. GO enrichment analysis of up-regulated genes was performed using Blast2GO which uses the Fisher’s Exact Test in combination with the Benjamini-Hochberg correction for multiple testing. A *P*-value cut-off of 0.05 was used to identify significant enriched GO terms.

## Supplementary Information

**Additional file 1: Figure S1.** Genomic profiling using short read data for the sequenced *Phytophthora cinnamomi* isolate (GKB4). **Figure S2.** GO enrichment analysis of up-regulated genes identified during infection in *Phytophthora cinnamomi*.

**Additional file 2: Supplementary Table 1.** Genome assembly statistics of GKB4 and other currently available genome sequences for *Phytophthora cinnamomi*. **Table S2.** RxLR effectors identified in the current version of *P. cinnamomi* genome following the method of McGowin and Fitzpatrick (2017). **Table S3.** Crinkler effectors identified in the current version of *P. cinnamomi* genome. **Table S4.** NLPs identified in the current version of *P. cinnamomi* genome. **Table S5.** CAZymes identified from the current version of *P. cinnamomi* genome. **Table S6.** Summary of RNA-Seq data generated in this study. **Table S7.** List of differentially expressed genes between mycelia and *in* *planta* infection as identified from DESeq2.

## Data Availability

The genomic sequence of *Phytophthora cinnamomi* (GKB4) has been deposited at DDBJ/EMBL/GenBank under the accession no. JAFJYM000000000. Whole genome and transcriptome data generated in this study has been deposited at the Sequence Read Archive under SRA accession no. PRJNA675400. Additional RNA-Seq data used in annotation of *P. cinnamomi* genome was obtained from the European Nucleotide Archive under accession no. ERR863673. Genome sequence data for two additional *P. cinnamomi* isolates (DU054 and WA94) used in ploidy analysis were obtained from SRA database under accession no. SRR6129791 and SRR6129792. The avocado genome (Hass cultivar) used for RNA Seq reference read mapping was obtained from GenBank genome database under accession no. SDSS00000000.

## Abbreviations

AA: Auxiliary activity
BLAST: Basic Local Alignment Search Tool
BUSCO: Benchmarking Universal Single-Copy Orthologs
CAZyme: Carbohydrate-active enzyme
CBM: Carbohydrate-binding module
CE: Carbohydrate esterase
CRN: Crinkler
CWDE: Cell wall degrading enzyme
DPI: Days post inoculation
EM: Expectation-Maximization
FDR: False discovery rate
GH: Glycoside hydrolase
GMM: Gaussian Mixture Model
GO: Gene ontology
HMM: Hidden Markov Model
NLP: Necrosis-inducing protein
noCat: No category
PL: Polysaccharide lyase
TE: Transposable element
