# Supplementary material for: Genome of the destructive oomycete Phytophthora cinnamomi provides insights into its pathogenicity and adaptive potential
Source: BMC Genomics. 2021 Apr 26;22:302. doi: 10.1186/s12864-021-07552-y (PMC8074420; doi:10.1186/s12864-021-07552-y)
Supplement: Supplementary file 1 — Additional file 1: Figure S1. Genomic profiling using short read data for the sequenced Phytophthora cinnamomi isolate (GKB4). Figure S2. GO enrichment analysis of up-regulated genes identified during infection in Phytophthora cinnamomi. [file 12864_2021_7552_MOESM1_ESM.docx]

**
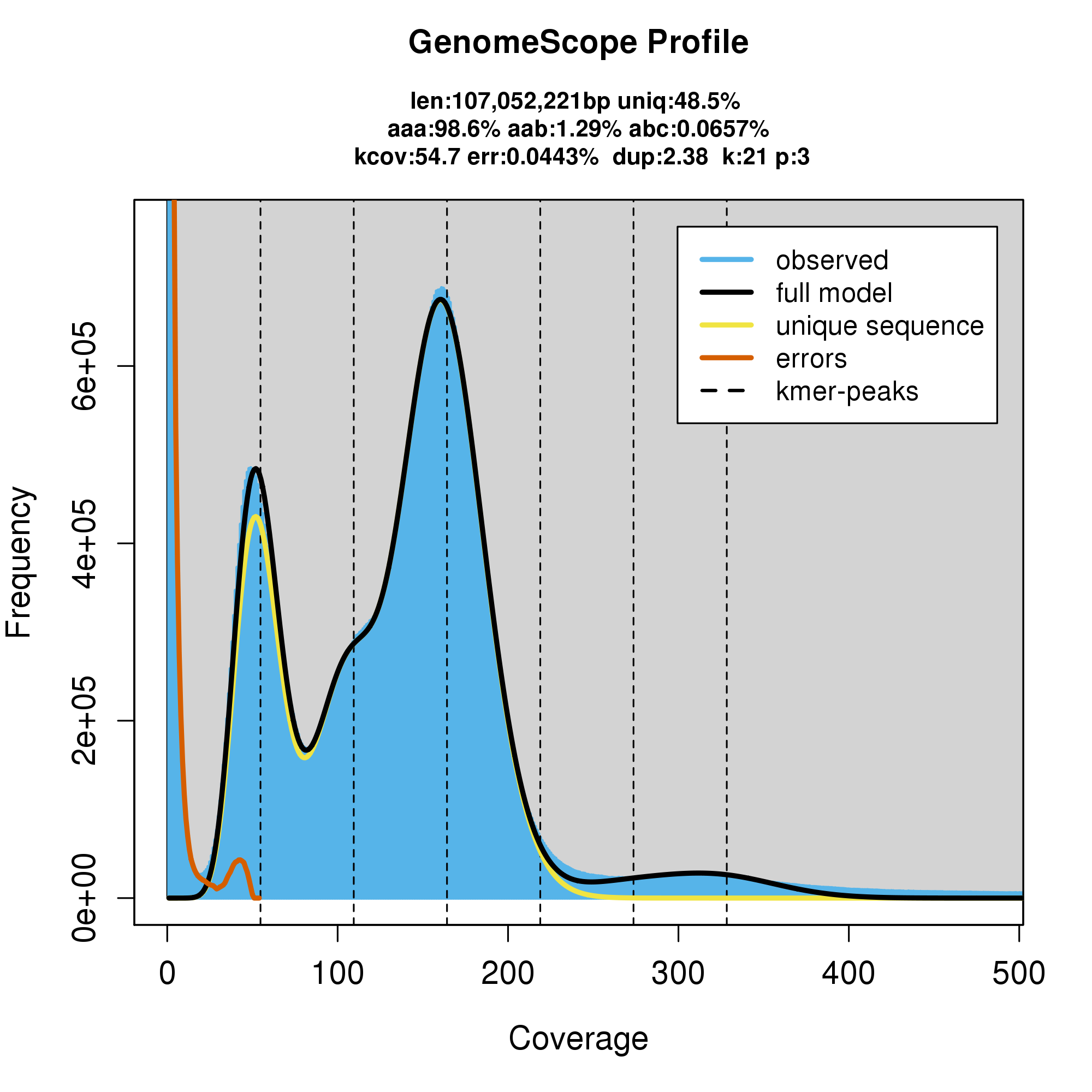
Figure S1.** Genomic profiling using short read data for the sequenced *Phytophthora cinnamomi* isolate (GKB4). The isolate was identified to be triploid (p:3) with an estimated genome size of 107 Mb and 1.36% genome-wide heterozygosity.

**
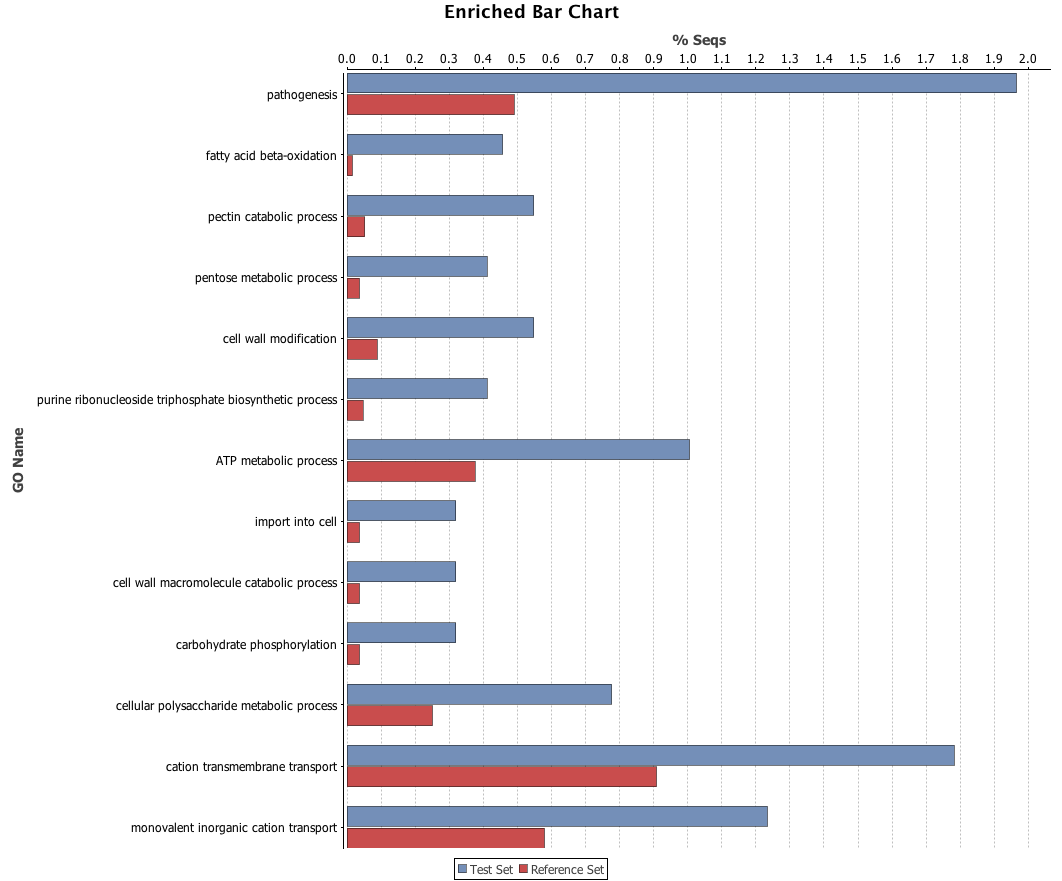
**
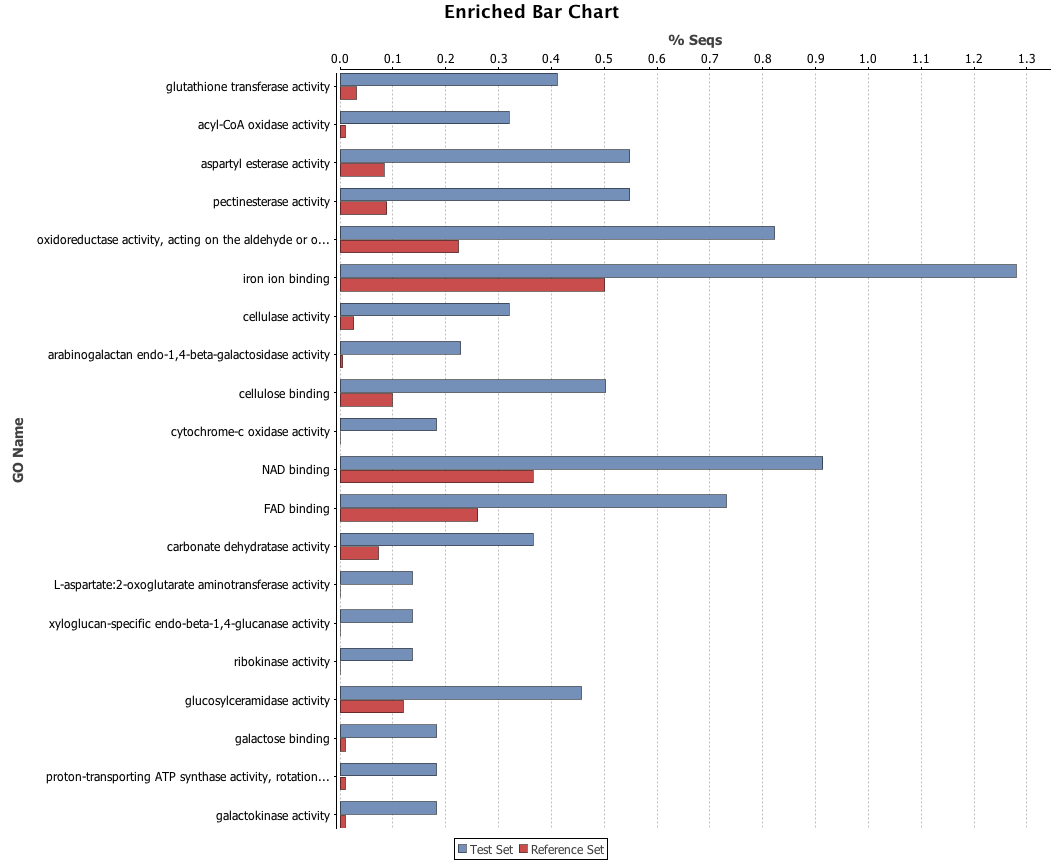


**Figure S2.** Bar charts showing the results of GO enrichment analysis of up-regulated genes identified during infection in *Phytophthora cinnamomi*. The results were visualized for biological processes (left) and molecular functions (right). Test Set consisted of genes identified to be upregulated during infection. Reference Set consisted of the rest of the genes from the genome. The X-axis indicates the percentage of genes counted.
